# Effects of metabolic and organ function factors on the efficacy of radioactive iodine therapy for hyperthyroidism

**DOI:** 10.3389/fendo.2025.1568699

**Published:** 2025-06-10

**Authors:** Yue Hu, Shan Liu, Xiaoliang Xiong, Lixing Wang, Yinlong Zhao

**Affiliations:** Department of Nuclear Medicine, The Second Hospital of Jilin University, Changchun, China

**Keywords:** hyperthyroidism, radioactive iodine treatment, iodine nutritional status, vitamin D, renal function

## Abstract

**Introduction:**

Recent studies suggest that metabolic and organ function indicators could influence radioactive iodine treatment (RAIT) efficacy in hyperthyroid patients, but their relationships with thyroid function remain unclear.

**Objective:**

This study aims to explore the impact of these markers on RAIT efficacy and thyroid function.

**Method:**

A total of 135 hyperthyroid patients undergoing RAIT were enrolled, with biomarkers collected at baseline and 1, 3, and 6 months posttreatment. These included thyrotropin receptor antibody (TRAb), free triiodothyronine (FT3), free thyroxine (FT4), thyroid-stimulating hormone (TSH), urinary iodine concentration (UIC), serum iodine concentration (SIC), 25-hydroxyvitamin D, alanine aminotransferase, aspartate aminotransferase, and serum creatinine (Scr). We mainly focus on the outcomes of the impact of metabolic and organ function markers on RAIT efficacy and their correlation with changes in thyroid function.

**Results:**

Logistic regression identified age as a predictor of RAIT efficacy (OR = 0.957, *p* = 0.025), with older patients less likely to achieve clinical improvement. Scr showed borderline significance (*p* = 0.049). Early after treatment, SIC was positively correlated with TSH (*r* = 0.204, *p* = 0.018), whereas UIC and SIC were significantly negatively correlated with TSH at 6 months during treatment (*p* < 0.05). In addition, higher Scr levels were consistently and significantly associated with lower FT3 and FT4 and higher TSH at multiple time points (*p* < 0.05).

**Conclusion:**

Age was a relative factor influencing RAIT efficacy, while iodine nutritional status exhibited dynamic, time-dependent effects on TRAb and TSH. Elevated Scr promotes reductions in FT3 and FT4 and an increase in TSH.

## Introduction

Thyrotoxicosis is a clinical syndrome characterized by an excess of circulating thyroid hormones within the body (1). The most prevalent cause of this condition is hyperthyroidism, an endocrine disorder arising from the overproduction and secretion of thyroid hormones (1, 2). The global prevalence of hyperthyroidism is estimated to range from 0.2% to 1.3% (3), with approximately 70% of these cases attributed to Graves’ hyperthyroidism (GH) (3). Currently, antithyroid drugs (ATDs), radioactive iodine treatment (RAIT), and thyroidectomy are the primary treatment options for hyperthyroidism, particularly in cases of GH (3). The initial treatment strategy for hyperthyroidism frequently involves the use of ATDs. However, the long-term effectiveness of ATDs is limited, with recurrence rates reaching up to 50%, and their use may be accompanied by adverse effects, including hepatotoxicity (4, 5). While thyroidectomy offers a potentially effective solution, its irreversible nature often results in permanent hypothyroidism or complications such as weight gain (3, 6). Conversely, RAIT has emerged as the preferred treatment modality for certain patients, particularly in North America, due to its noninvasive nature, sustained efficacy, and comparatively low recurrence rate (7, 8).

Radioactive iodine (RAI) is transported to thyroid tissue via the sodium/iodide symporter (9). Once accumulated, it emits beta radiation that generates free radicals and induces deoxyribonucleic acid damage, inducing cytotoxicity that destroys thyroid tissue and reduces hormone synthesis (10). For patients with GH who have a poor response to ATDs or experience relapse, RAIT has shown significant advantages (11). However, despite the long-term efficacy achieved in most patients, a minority may still develop adverse effects, such as hypothyroidism (12, 13). Although RAIT primarily targets thyroid tissue, other cells can also accumulate RAI. For example, salivary gland uptake may result in glandular injury (14, 15), and RAI can also activate thyroid‐specific autoimmune responses, evidenced by changes in thyrotropin receptor antibody (TRAb) and antithyroglobulin antibody levels and transient elevations of inflammatory cytokines, potentially leading to new‐onset or worsened thyroid‐associated ophthalmopathy (16). Moreover, the potential carcinogenic risk of RAIT cannot be completely excluded (16, 17). Therefore, close posttreatment monitoring and long-term follow-up are essential to ensure its safety and efficacy.

A growing body of research indicates that the effectiveness of RAIT may be modulated by multiple interacting factors, including RAI dose, thyroid volume, age, and sex (16). Furthermore, iodine nutritional status emerges as a pivotal determinant influencing RAIT outcome. To optimize thyroid uptake of RAI, patients are frequently recommended to follow a low-iodine diet (LID) before treatment (12, 18, 19). However, recent evidence suggests that there is no significant association between short-term LID and the effects of RAIT (20, 21). Although lower urinary iodine concentration (UIC) can increase RAI uptake, it does not significantly improve the treatment’s success rate, thereby questioning the necessity of LID (21). Compared to UIC, serum iodine concentration (SIC) is a more immediate indicator of thyroid iodine utilization and metabolic dynamics (22, 23). Studies have shown that both excessively low and high SIC may lead to thyroid dysfunction, thereby impacting the effectiveness of RAIT (23).

The immunomodulating effects of vitamin D (vit D) have been shown to improve treatment outcomes in patients with GH (24–26). Studies have indicated vit D deficiency is associated with a high prevalence of thyroid autoimmune disease, especially during RAIT. Supplementation with vit D has a potential positive impact on reducing hyperthyroidism recurrence (24), but further studies are necessary to confirm the specific role in RAIT (13).

The liver is integral to the metabolism and conversion of thyroid hormones, and impaired liver function may affect the restoration and regulation of thyroid hormone homeostasis (27). Likewise, the kidneys serve as the principal organs for the excretion of RAI (28). Renal dysfunction may prolong the retention time of RAI within the body, thereby consequently affecting the therapeutic efficacy (28). Since many patients may have pre-existing liver and kidney damage caused by prior medication treatments before undergoing RAIT, the functional status of these organs may indirectly impact the efficacy and safety of RAIT by altering the processes of RAI uptake, distribution, and clearance (27, 28).

While previous studies have explored the relationship between RAIT and metabolic as well as organ functional status, there remains a paucity of research directly examining how these factors influence the efficacy of RAIT. This study aims to systematically evaluate the relationship between iodine nutritional status, vit D levels, liver function, kidney function, and the effectiveness of RAIT in the treatment of hyperthyroidism. It seeks to analyze the potential factors that may affect the therapeutic outcomes of RAIT.

## Materials and methods

### Patients selection

This study was approved by the Medical Ethics Committee of the Second Hospital of Jilin University (Approval No.: 2023-231). All participants provided informed consent prior to their involvement in the study. We collected data from patients who received RAIT at the Department of Nuclear Medicine of the Second Hospital of Jilin University between 12 November 2023 and 1 May 2024. Eligible patients meeting the following criteria were included: (1) diagnosed with hyperthyroidism; (2) suitable for and receiving RAIT for the first time; and (3) followed up at 1, 3, or 6 months after treatment. Exclusion criteria were as follows: (1) a history of neoplasm; (2) patients with comorbid nonhyperthyroid thyroid disorders, psychiatric disorders, or pregnancy/lactation status (3) structural damage to vital organs such as the liver and kidney, or hepatic and renal dysfunction; and (4) incomplete follow-up information. All patients included in the study provided written informed consent for RAIT and the associated procedures.

### Data collection

We collected biomarker data from patients at baseline (before RAIT) and at 1, 3, and 6 months after treatment. The biomarkers included thyroid function (TRAb, free triiodothyronine [FT3], free thyroxine [FT4], and thyroid-stimulating hormone [TSH]), as well as UIC, SIC, 25-hydroxyvitamin D (25[OH]D), alanine aminotransferase (ALT), aspartate aminotransferase (AST), and serum creatinine (Scr). Thyroid function (TRAb, FT3, FT4, and TSH) was measured by electrochemiluminescence immunoassay. The detection ranges for these markers were as follows: TRAb, up to 40 IU/L; FT3, up to 50 pmol/L; FT4, up to 100 pmol/L; and TSH, from a minimum of 0.005 mIU/L to a maximum of 100 mIU/L. UIC and SIC were determined using the DAT-20SG and DAT-500 systems (Silky-Road Medical Technology, Changsha, China), respectively. Both systems employ arsenic-cerium catalytic spectrophotometry; additionally, the DAT-500 protocol incorporates a cold-digestion step based on international guidelines for serum iodine analysis, achieving an analytical accuracy error of ≤ 5%, an intra-assay coefficient of variation (CV) ≤ 2%, and an interassay CV ≤ 6%. 25 (OH) D was analyzed by chemiluminescence. ALT and AST were determined using the IFCC method without the addition of P5P, while Scr was assessed via the creatinine oxidase assay. Detailed information on data analysis methods and analytical instruments is provided in **Supplementary Table S1**.

### Statistical analysis

Normality tests were conducted on the collected data to assess whether they followed a normal distribution. Based on the results of the normality tests, we used either paired sample t-tests or Wilcoxon signed-rank tests, along with one-way analysis of variance (ANOVA) or Friedman tests, to evaluate significant changes in thyroid function at different time points before and after RAI treatment. ANOVA or Kruskal–Wallis tests were used to assess the differences in biomarkers across groups with varying treatment outcomes. For biomarkers with significant differences, logistic regression analysis was applied to identify outcome-related predictors. To analyze the potential relationships between biomarkers and thyroid function at various time points, we used Pearson or Spearman bivariate correlation analyses, depending on data distribution. A linear mixed model (LMM) was used to further analyze the effects of time and biomarkers on thyroid function and its rate of change. The model incorporated fixed effects, and parameter estimation was performed using maximum likelihood estimation, with individual patients treated as random effects to control for inter-individual variability. Residual analysis was conducted to verify model assumptions. All statistical analyses were performed using SPSS 29.0 (IBM Corp, Armonk, NY, USA), with a two-sided *p* < 0.05 considered statistically significant.

## Results

### Patient clinical characteristics

**Figure 1:**
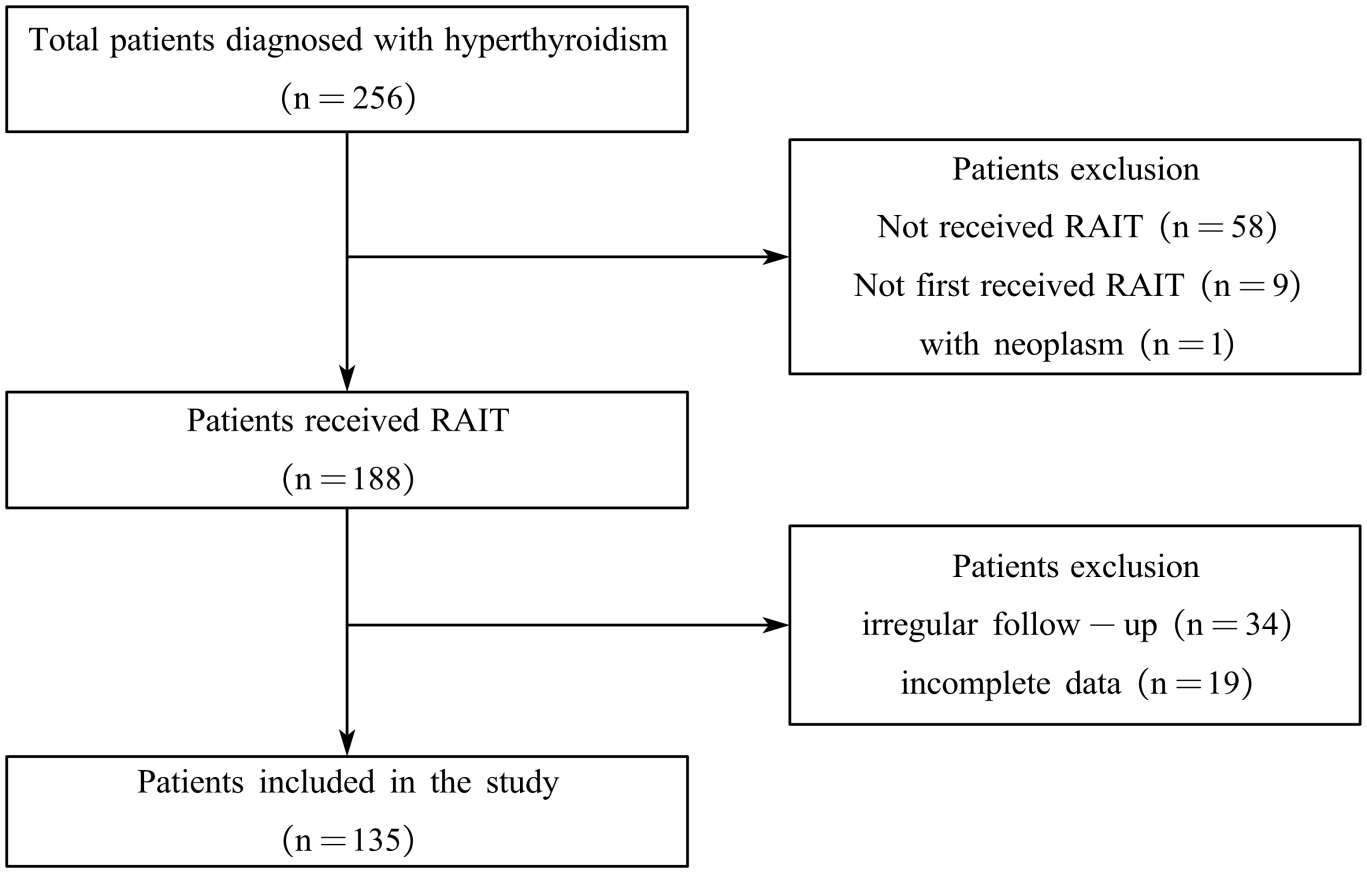
Flowchart of the enrollment and exclusion of study participants. RAIT, radioactive iodine treatment.

As shown in **Figure 1**, of the 256 patients with hyperthyroidism who underwent RAIT, 135 met our inclusion criteria by completing the required follow-up and were thus included in the study. The characteristics of the included patient cohort are summarized in **Table 1**. Overall, the median age was 45 years (range: 21–72), and 61.48% (83 of 135) were women. Prior to RAIT, 77.04% (104 of 135) of patients had received drug therapy, with 38.46% (40 of 104) experiencing relapse after treatment. Palpitations (82.22%) and tremors (60.00%) were the most common symptoms observed before RAIT. Additionally, 65.19% (88 of 135) of the patients had a history of smoking, and 24.44% (33 of 135) had a history of consuming iodine-rich foods. The median total thyroid weight was 37.9 g (range: 10.2–88.9), with median weights of the left and right lobes being 19 g (range: 4.4–41.9) and 19 g (range: 4.8–47.2), respectively. The median radioactive iodine uptake (RAIU) was 54.3% (range 22.3%–99.0%).

**Table 1:** Patient baseline characteristics.

Characteristic	Total (*N* = 135)
Median age (range; years)	45 (21–72)
Sex (*n*; %)	
Male	52 (38.52)
Female	83 (61.48)
Smoker (*n*; %)	88 (65.19)
Median disease duration (range; years)	3 (0–30)
Prior therapy (*n*; %)	
Untreated	31 (22.96)
Drug therapy	104 (77.04)
ATD/ATD and LT4	99/5 (95.19/4.81)
Surgery	1 (0.74)
Relapse after treatment (*n*; % of drug therapy)	40 (38.46)
Symptomatic (*n*; %)	
Palpitations	111 (82.22)
Tremor	81 (60.00)
Exophthalmos	65 (48.15)
Iodine-rich food consumption (*n*; %)	33 (24.44)
Thyroid median weight (range; g)	
Left lobe weight	19 (4–41.9)
Right lobe weight	19 (4.8–47.2)
Total weight	37.9 (10.2–88.9)
Median RAIU (%; range)	54.3 (22.3–99.0)

*ATD*, antithyroid drug; *LT4*, levothyroxine; *RAIU*, radioactive iodine uptake.

To systematically assess the dynamic effects of RAIT on biomarkers in patients, we collected and analyzed biomarkers at baseline and at 1, 3, and 6 months posttreatment (**Table 2**). Due to the lower detection limit of the laboratory assay, baseline TSH levels for most patients were recorded as 0.005 mIU/L (0.005, 0.005). TRAb levels increased from a baseline of 17.8 IU/L (8.7, 36.0) to 40.0 IU/L (25.8, 40.0) at 6 months. TSH also rose significantly to 5.160 mIU/L (0.005, 37.600) at 6 months. FT3 and FT4 levels exhibited a progressive decline (FT3: baseline, 27.4 pmol/L [15.1, 42.1], 6 months posttreatment, 5.1 pmol/L [3.0, 8.2]; FT4: baseline, 75.8 pmol/L [42.5, 100.0], 6 months posttreatment, 16.8 pmol/L [7.0, 25.2]). Iodine nutritional status showed an initial increase followed by a decrease (e.g., SIC: baseline, 183.1 µg/L [143.7, 241.0]; 1 month posttreatment, 200.9 µg/L [149.5, 253.4]; 6 months posttreatment, 126.7 µg/L [98.4, 178.3]). In contrast, the interquartile ranges of 25(OH)D, ALT, AST, and Scr exhibited relatively minor changes across all time points before and after treatment.

**Table 2:** Baseline and posttreatment biomarker characteristics by follow-up period.

Category	Biomarker	Baseline (median [Q1, Q3])	1 Month posttreatment (median [Q1, Q3])	3 Months posttreatment (median [Q1, Q3])	6 Months posttreatment (median [Q1, Q3])
Thyroid function	TRAb (IU/L)^a^	17.8 [8.7, 36.0]	21.5 [12.8, 34.6]	36.3 [26.9, 40.0]	40.0 [25.8, 40.0]
	FT3 (pmol/L)^b^	27.4 [15.1, 42.1]	8.2 [5.5, 20.3]	5.0 [2.6, 8.5]	5.1 [3.0, 8.2]
	FT4 (pmol/L)^c^	75.8 [42.5, 100.0]	26.9 [18.6, 48.5]	15.8 [7.2, 24.6]	16.8 [7.0, 25.2]
	TSH (mIU/L)^d^	0.005 [0.005, 0.005]	0.005 [0.005, 0.005]	4.270 [0.005, 27.100]	5.160 [0.005, 37.600]
Iodine nutrition	UIC (μg/L)	103.8 [66.4, 187.6]	221.6 [142.6, 322.0]	152.4 [94.7, 194.6]	114.0 [76.4, 165.8]
	SIC (μg/L)	183.1 [143.7, 241.0]	200.9 [149.5, 253.4]	169.1 [132.2, 225.0]	126.7 [98.4, 178.3]
Vitamin D	25(OH)D (nmol/L)	42.30 [28.9, 57.8]	52.0 [36.7, 61.4]	47.2 [36.4, 56.0]	49.2 [42.6, 56.4]
Liver function	ALT (U/L)	26.0 [21.0, 42.0]	26.0 [19.0, 36.0]	24.0 [17.0, 31.0]	25.0 [18.0, 34.0]
	AST (U/L)	25.0 [19.0, 35.0]	25.0 [19.0, 32.0]	25.0 [18.0, 33.0]	25.0 [19.0, 34.0]
Kidney function	Scr (μmol/L)	44.0 [37.0, 58.0]	49.6 [40.0, 60.0]	56.0 [45.0, 67.0]	56.0 [47.0, 69.3]

Median [Q1, Q3]: values are presented as the median with the first quartile (Q1) and third quartile (Q3) in brackets.

*TRAb*, thyrotropin receptor antibody; *FT3*, free triiodothyronine; *FT4*, free thyroxine; *TSH*, thyroid-stimulating hormone; *UIC*, urinary iodine concentrations; *SIC*, serum iodine concentrations; *25(OH)D*, 25-hydroxyvitamin D; *ALT*, alanine aminotransferase; *AST*, aspartate aminotransferase; *Scr*, serum creatinine.

a TRAb has an upper detection limit of 40 IU/L in the laboratory assay; values exceeding this threshold are reported as 40 IU/L.

b FT3 has an upper detection limit of 50 pmol/L in the laboratory assay; values exceeding this threshold are reported as 50 pmol/L.

c FT4 has an upper detection limit of 100 pmol/L in the laboratory assay; values exceeding this threshold are reported as 100 pmol/L.

d TSH has a lower detection limit of 0.005 mIU/L in the laboratory assay; values below this threshold are reported as 0.005 mIU/L.

Normality test results indicated that most biomarkers did not follow a normal distribution at various time points (**Supplementary Table S2**). Based on this result, nonparametric tests were applied. The results of the Wilcoxon signed-rank test and the Friedman test showed significant changes in TRAb, FT3, FT4, TSH, UIC, SIC, 25(OH)D, and Scr across different time points before and after treatment (*p* < 0.05), whereas no significant changes were observed for ALT and AST between time points (**Supplementary Tables S3, S4**).

### Analysis of factors associated with the efficacy of RAIT

Efficacy was categorized according to the thyroid function and clinical manifestations of the patients after 6 months of treatment. Clinical cure was defined as the complete resolution of hyperthyroid symptoms and signs, with FT3, FT4, and TSH levels returning to normal, or the appearance of hypothyroid features (FT3 or FT4 below normal and TSH above normal). Improvement was defined as a reduction in hyperthyroid symptoms and partial resolution of signs, with significant reductions in FT3 and FT4, though not within normal limits. Ineffective was classified as the lack of improvement or worsening of hyperthyroid symptoms and signs following treatment. A significant change in thyroid function was defined as a posttreatment change of 20% or more relative to pretreatment levels over the 6-month period. Among the patients, 56 achieved clinical cure, 69 showed improvement, and 10 were ineffective (**Supplementary Table S5**). Patients in the ineffective group had lower FT3 and FT4 levels before treatment compared to the clinical cure and improvement groups, while their TRAb levels were higher than those in the other two groups.

**Figure 2:**
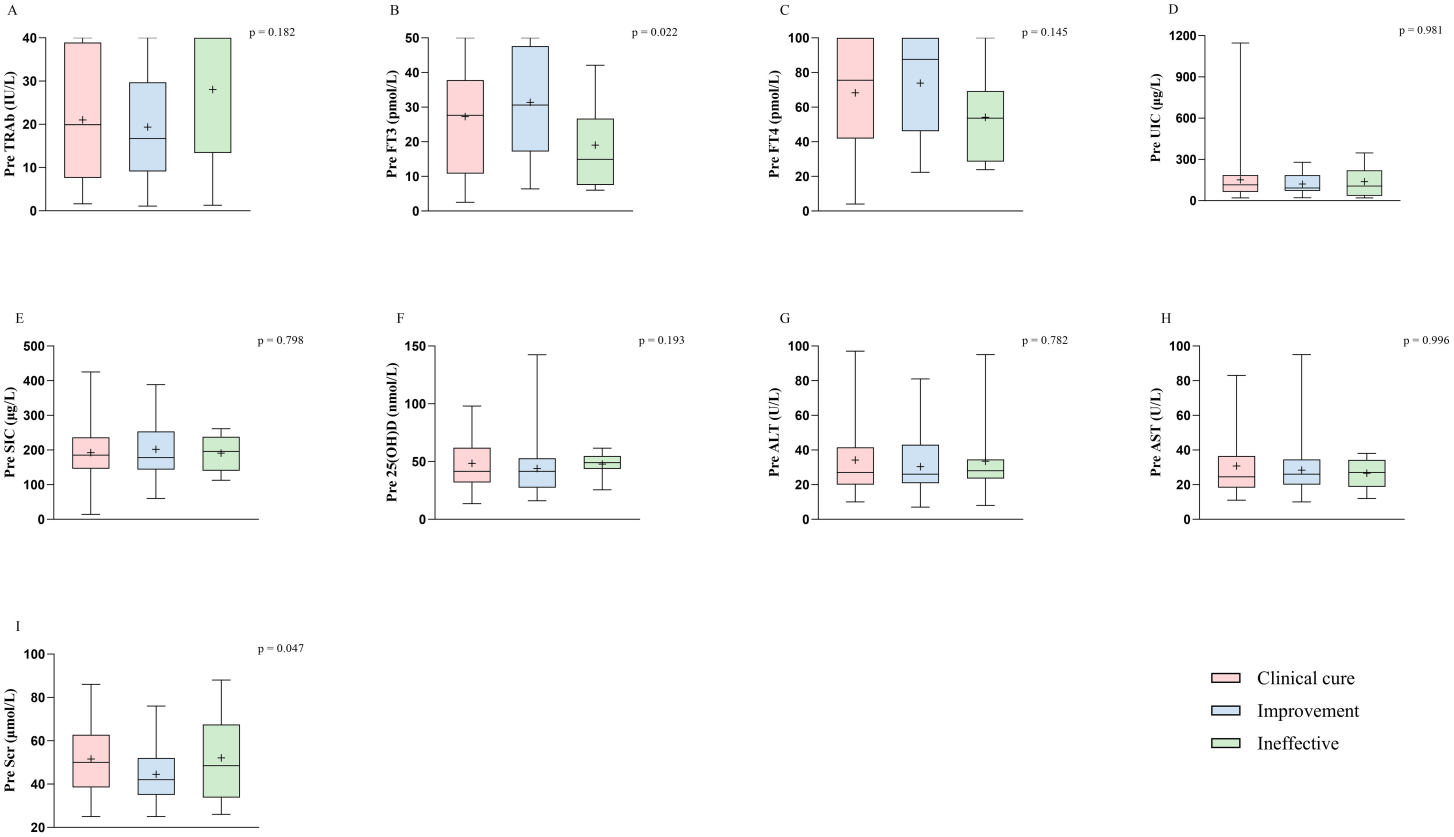
Biological marker characteristics prior to treatment in different efficacy groups at 6 months after RAIT. The biological markers include nine variables: TRAb **(A)**, FT3 **(B)**, FT4 **(C)**, UIC **(D)**, SIC **(E)**, 25(OH)D **(F)**, ALT **(G)**, AST **(H)**, and Scr **(I)**. TSH is not included because most patients had levels close to 0.005 before treatment. *p* < 0.05 was considered to indicate significant differences among the clinical cure, improvement, and ineffective groups. RAIT, radioactive iodine treatment; Pre, pretreatment; TRAb, thyrotropin receptor antibody; FT3, free triiodothyronine; FT4, free thyroxine; TSH, thyroid-stimulating hormone; UIC, urinary iodine concentrations; SIC, serum iodine concentrations; 25(OH)D, 25-hydroxyvitamin D; ALT, alanine aminotransferase; AST, aspartate aminotransferase; Scr, serum creatinine.

Patient characteristics and baseline biomarkers between groups were included in nonparametric tests. Using the Kruskal–Wallis test to evaluate the significance of age and biomarkers among different groups, we identified significant differences in age, baseline FT3, and baseline Scr across efficacy (**Figure 2**; **Supplementary Table S5**; age, *p* = 0.044; FT3, *p* = 0.022; Scr, *p* = 0.047). These factors were subsequently incorporated into a multivariate logistic regression model to analyze their influence on efficacy in hyperthyroid patients at 6 months after RAIT. The results indicated that age was an independent predictor for clinical cure and improvement (odd ratio (OR) = 0.957, 95% confidence interval (CI) = 0.921–0.994, *p* = 0.025), suggesting a significant negative correlation between age and efficacy (**Table 3**). Specifically, older patients were less likely to achieve a clinical cure. Meanwhile, we observed that pretreatment Scr demonstrated statistical significance between clinical cure and improvement (*p* = 0.049), and this significance was borderline (**Table 3**). Furthermore, with an OR of 0.970 and the upper limit of the 95% CI approaching 1.0, these findings suggest that while Scr maintains a correlation with treatment outcome, its independent influence might be relatively weak. Pretreatment FT3 did not demonstrate a significant independent predictive role between clinical cure and improvement. Additionally, no significant independent predictors, including sex, age, and pretreatment FT3 and Scr, were identified between the clinical cure and ineffective treatment groups.

**Table 3:** Multivariate logistic regression analysis of influencing factors at 6 months after RAIT in patients with hyperthyroidism.

Influencing factors	Improvement vs. clinical cure		Ineffective vs. clinical cure	
	OR (95% CI)	*P*-value	OR (95% CI)	*P*-value
Age	**0.957 (0.921, 0.994)**	**0.025**	0.954 (0.888, 1.025)	0.198
Pre FT3	0.999 (0.971, 1.028)	0.953	0.947 (0.892, 1.004)	0.070
Pre Scr	**0.970 (0.941, 1.000)**	**0.049**	0.980 (0.930, 1.032)	0.446

*RAIT*, radioactive iodine treatment; *95% CI*, 95% confidence interval; *Pre*, pretreatment; *FT3*, free triiodothyronine; *Scr*, serum creatinine. Bold values indicate statistical significance (p < 0.05).

### Correlation between metabolic and organ function markers and thyroid function

**Figure 3:**
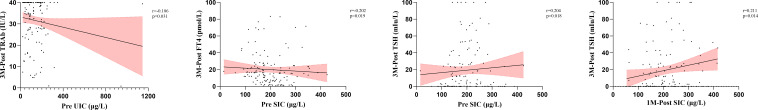
Analysis of the correlation between iodine nutritional status and thyroid function after RAIT. RAIT, radioactive iodine treatment; Pre, pretreatment; 3M-Post, 3 months posttreatment; TRAb, thyrotropin receptor antibody; FT3, free triiodothyronine; FT4, free thyroxine; TSH, thyroid-stimulating hormone; UIC, urinary iodine concentrations; SIC, serum iodine concentrations.

**Figure 4:**
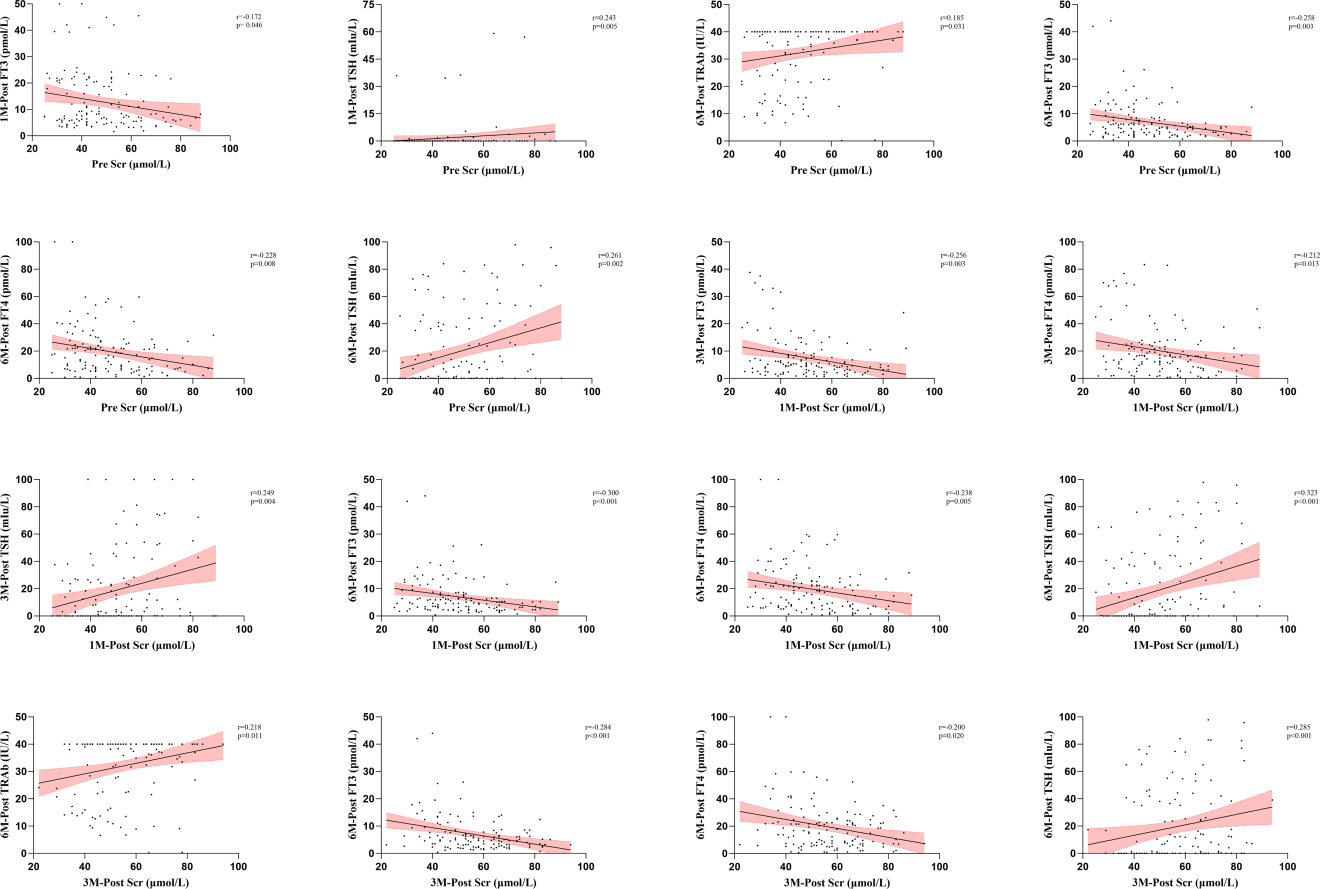
Analysis of the correlation between Scr and thyroid function after RAIT. RAIT, radioactive iodine treatment; Pre, pretreatment; 1M-Post, 1 month posttreatment; 3M-Post, 3 months posttreatment; 6M-Post, 6 months posttreatment; TRAb, thyrotropin receptor antibody; FT3, free triiodothyronine; FT4, free thyroxine; TSH, thyroid-stimulating hormone; Scr, serum creatinine.

Spearman correlation analysis was used to evaluate the potential impacts of metabolic and organ function markers on thyroid function at different time points. In this analysis, we identified 28 significant correlations between metabolic and organ function indicators before and after treatment and thyroid function after treatment (**Figures 3**, **4**, **Supplementary Table S6**). Specifically, there were 1, 3, 3, 5, and 16 correlations between UIC, SIC, ALT, AST, and Scr and thyroid functional status, respectively. The correlation between UIC and thyroid function was relatively weak. Pretreatment UIC exhibited a negative correlation with TRAb at 3 months posttreatment (**Figure 3**, r = − 0.186, *p* = 0.031). In contrast, SIC demonstrated more complex associations. Pretreatment SIC showed a negative correlation with FT3 (*r* = − 0.202, *p* = 0.019) and a positive correlation with TSH (*r* = 0.204, *p* = 0.018) at 3 months posttreatment (**Figure 3**). Additionally, at 1 month posttreatment, SIC remained positively correlated with TSH at 3 months posttreatment (*r* = 0.211, *p* = 0.014), suggesting a relatively strong influence of SIC on TSH at 3 months posttreatment. The relationship between Scr and thyroid function was the most pronounced and exhibited consistent trends across multiple time points. Pretreatment Scr was strongly negatively correlated with FT3 (*r* = − 0.258, *p* = 0.003) and FT4 (*r* = − 0.228, *p* = 0.008) at 6 months posttreatment, whereas it was positively correlated with TSH (*r* = 0.261, *p* = 0.002). This trend persisted after treatment, particularly at 3 months posttreatment, where Scr remained negatively correlated with FT3 (*r* = − 0.284, *p* < 0.001) and FT4 (*r* = − 0.200, *p* = 0.020) and positively correlated with TSH (*r* = 0.285, *p* < 0.001). These correlations were consistent across several time points (**Figure 4**).

During the treatment process, no significant correlation was found between 25(OH)D and posttreatment thyroid function (**Supplementary Table S6**). However, ALT and AST exhibited some associations with thyroid function, mainly in the pre-treatment period and the early stage of treatment. For instance, pretreatment ALT showed a positive correlation with FT3 (*r* = 0.174, *p* = 0.043) and FT4 (*r* = 0.195, *p* = 0.024) at 1 month posttreatment (**Supplementary Table S6**). Additionally, pretreatment AST was positively correlated with TRAb at 3 months posttreatment (**Supplementary Table S6**, *r* = 0.209, *p* = 0.015). Notably, these associations did not persist at later time points. Results from nonparametric tests indicated that ALT and AST did not exhibit significant differences throughout the treatment process (**Supplementary Tables S3, S4**). While these indicators showed correlation with thyroid function at specific time points, they may not constitute the primary influencing factors for alterations in thyroid function.

### Longitudinal impact of metabolic and organ function markers on treatment

In the LMM analysis, incorporating time as a repeated measure and individual patients as random effects, we observed that multiple metabolic and organ function markers were significantly associated with changes in thyroid function before and after treatment (**Table 4**). The analysis results showed that both UIC and SIC were significantly negatively correlated with TRAb and TSH. Specifically, the negative correlations between SIC and TRAb (*β* = − 0.041, *p* < 0.001) as well as TSH (*β* = − 0.059, *p* < 0.001) were stronger than those between UIC and TRAb (*β* = − 0.010, *p* = 0.002) and TSH (*β* = − 0.020, *p* = 0.002), and these negative associations were more pronounced. Additionally, SIC showed significant positive correlations with FT3 and FT4 (FT3: *β* = 0.017, *p* = 0.022; FT4: *β* = 0.060, *p* = 0.003), but no similar association was observed between UIC and FT3. These findings suggest that iodine nutritional status has a significant regulatory effect on thyroid function, particularly with regard to TRAb and TSH, following RAIT, and this effect may change with the progression of treatment. Through repeated measures analysis, Scr was found to be significantly negatively correlated with both FT3 (*β* = − 0.460, *p* < 0.001) and FT4 (*β* = − 1.029, *p* < 0.001), while it was significantly positively correlated with TRAb (*β* = 0.102, *p* = 0.018) and TSH (*β* = 0.651, *p* < 0.001). This further indicated that renal function exerts a significant and persistent influence on thyroid function changes before and after RAIT.

**Table 4:** Effects of iodine nutritional status, vitamin D, liver function, and renal function-related biomarkers on thyroid function assessed using LMM analysis.

Variables	Mean ± SE	UIC	SIC	25(OH)D	ALT	AST	Scr
TRAb	Pre = 20.68 ± 1.17	***β* = − 0.010**	***β* = − 0.041**	*β* = 0.008	*β* = 0.014	***β* = 0.109**	***β* = 0.102**
	1M = 22.80 ± 1.12	***p* = 0.002**	***p* < 0.001**	*p* = 0.454	*p* = 0.688	***p* < 0.001**	***p* = 0.018**
	3M = 31.62 ± 0.93						
	6M = 32.34 ± 0.97						
FT3	Pre = 28.75 ± 1.30	*β* = − 0.008	***β* = 0.017**	*β* = − 0.010	***β* = 0.126**	*β* = 0.024	***β* = − 0.460**
	1M = 12.91 ± 0.93	*p* = 0.050	***p* = 0.022**	*p* = 0.509	***p* = 0.002**	*p* = 0.497	***p* < 0.001**
	3M = 7.49 ± 0.66						
	6M = 6.92 ± 0.57						
FT4	Pre = 70.09 ± 2.56	*β* = − 0.007	***β* = 0.060**	*β* = − 0.025	***β* = 0.298**	*β* = 0.045	***β* = − 1.029**
	1M = 36.37 ± 2.21	*p* = 0.442	***p* = 0.003**	*p* = 0.442	***p* < 0.001**	*p* = 0.567	***p* < 0.001**
	3M = 20.02 ± 1.60						
	6M = 19.49 ± 1.47						
TSH	Pre = 0.04 ± 0.19	***β* = − 0.020**	***β* = − 0.059**	*β* = 0.000	*β* = 0.032	***β* = 0.232**	***β* = 0.651**
	1M = 1.89 ± 0.75	***p* = 0.002**	***p* < 0.001**	*p* = 0.986	*p* = 0.606	***p* < 0.001**	***p* < 0.001**
	3M = 19.21 ± 2.36						
	6M = 19.60 ± 2.32						

*LMM*, linear mixed model; *SE*, standard error; *Pre*, pretreatment; *1M*, 1 Month posttreatment; *3M*, 3 months posttreatment; *6M*, 6 months posttreatment; *TRAb*, thyrotropin receptor antibody; *FT3*, free triiodothyronine; *FT4*, free thyroxine; *TSH*, thyroid-stimulating hormone; *UIC*, urinary iodine concentrations; *SIC*, serum iodine concentrations; *25(OH)D*, 25-hydroxyvitamin D; *ALT*, alanine aminotransferase; *AST*, aspartate aminotransferase; *Scr*, serum creatinine. Bold values indicate statistical significance (p < 0.05).

Throughout the treatment course, no significant correlation was observed between 25(OH)D and thyroid function alterations. Although nonparametric tests revealed no significant intertemporal differences in ALT and AST, LMM analysis demonstrated potential associations between these parameters following RAIT. Notably, in the temporal dimension of multiple measurements, ALT and AST exhibited certain correlations with thyroid function fluctuations across different time points, suggesting possible time-dependent effects of thyroid function on ALT and AST.

During the residual analysis, we found that the residuals did not obey a normal distribution (**Supplementary Table S7**). This result may be related to the distribution characteristics of the data, potentially reflecting sample heterogeneity or the influence of some unaccounted variables. Nevertheless, considering that the LMM, after controlling for individual differences and time factors, still demonstrated significant associations between metabolic and organ function markers and thyroid function, we maintain that the analysis results are robust and reliable.

## Discussion

In recent years, RAIT has demonstrated significant advantages in treating hyperthyroidism and has increasingly been considered the preferred treatment option for patients (7, 8). Emerging findings underscore the complexity of factors that can impact RAIT efficacy and highlight the need for personalized treatment approaches to optimize outcomes for patients with hyperthyroidism. Thus far, factors affecting RAIT efficacy still remain a focal point of current clinical research.

In this study, 135 patients undergoing RAIT were enrolled. Data on TRAb, FT3, FT4, TSH, UIC, SIC, 25(OH)D, ALT, AST, and Scr were collected and analyzed at baseline and at 1, 3, and 6 months posttreatment, identifying four key findings. First, approximately 41.49% of patients achieved clinical cure following RAIT, and approximately 51.11% of patients showed improvement. Age was identified as a relative influencing factor for the efficacy of RAIT (**Table 3**). Second, the study revealed that iodine nutritional status significantly affected thyroid function changes after RAIT, and the extent of the effect varied over time (**Table 4**, **Supplementary Table S6**). In the early posttreatment period, higher iodine nutrition levels were more conducive to the recovery of thyroid function, while in the later stages, lower iodine levels were more beneficial for treatment efficacy. Third, within the normal range, relatively higher Scr levels were associated with a greater decrease in FT3 and FT4, as well as an increase in TSH (**Table 4**, **Supplementary Table S6**). Finally, data analysis revealed that changes in ALT and AST before and after RAIT were not significant (**Supplementary Tables S3, S4**), and 25(OH)D had little impact on the efficacy following RAIT (**Tables 3**, **4**, **Supplementary Table S6**).

In recent years, the advent of RAIT has markedly transformed the therapeutic approach to hyperthyroidism. Nonetheless, the influence of patient age on the efficacy of RAIT remains a subject of debate within the academic community. Certain studies propose that age does not significantly affect the efficacy of RAIT in individuals with GH, suggesting that younger patients generally exhibit lower success rates, a pattern that endures even after controlling for confounding variables (29–31). In contrast, other studies show a negative correlation between age and cure rates, with younger patients more likely to develop hypothyroidism, suggesting higher sensitivity to RAIT (32–34). Bonnema et al. proposed that these discrepancies may be attributed to differences in smoking habits, disease severity, medication use, and thyroid enlargement in younger patients (16). The results presented in this study suggest that age may serve as an independent predictor for clinical cure and improvement (OR = 0.957, *p* = 0.025). This also supports the finding that younger patients are more sensitive to RAI and are more likely to benefit from the treatment. However, in the comparative analysis of clinical cure and ineffectiveness, the independent predictive role of age did not reach statistical significance (OR = 0.954, *p* = 0.198). Therefore, although the conclusions from existing studies are inconsistent, our data suggest that age may serve as a relative predictor of treatment efficacy rather than an independent predictor. Additionally, our study also supports that younger patients are more likely to benefit from RAIT.

UIC is the predominant metric employed for evaluating the iodine nutritional status of populations; however, it is subject to variation due to several factors, including dietary iodine intake and fluid consumption (35, 36). In contrast, SIC is less affected by dietary factors and remains relatively stable (37). This aligns with the findings of our study, where SIC showed a more significant association with thyroid function compared to UIC (**Supplementary Table S6**) (23, 38). Although a LID is typically recommended prior to RAIT, recent studies on iodine nutrition status and RAIT efficacy indicate that a LID does not provide additional benefits for RAIT (20, 21). In our analysis, we observed a more complex relationship between iodine nutrition status and RAIT efficacy.

In the LMM with repeated measurements over 6 months after RAIT, both UIC and SIC showed significant negative correlations with TRAb and TSH. Notably, despite the expected therapeutic effects of RAIT to decrease TRAb and increase TSH, some studies have reported that TRAb may actually rise within 6 months of RAIT, which is associated with thyroid cell damage, antigen release, and autoimmune reactions (39–41). Based on this finding, we hypothesize that the dynamic regulation of iodine nutrition status may have a bidirectional impact on RAIT efficacy. Higher iodine nutritional status may help reduce TRAb; however, it could also lead to a further increase in thyroid hormone levels, thereby exacerbating the feedback inhibition on TSH. Lindgren et al. and Fang et al. also confirmed this viewpoint (39, 42).

Furthermore, the role of time factors in the relationship between iodine nutritional status and thyroid function parameters following RAIT deserves further exploration. The results indicate that a higher iodine nutritional status prior to treatment may contribute to a decrease in TRAb and an increase in TSH during the early phase posttreatment. In the LMM analysis, however, a lower iodine nutritional status throughout the follow-up period appears to be more favorable for the stabilization of thyroid function. This finding suggests that iodine nutritional status not only exhibits a bidirectional regulatory effect on thyroid function after RAIT but also has time-dependent characteristics. An elevated SIC at the onset of treatment may improve therapeutic outcomes by suppressing TRAb and increasing TSH levels. However, as treatment advances, the persistent feedback inhibition of TSH influenced by iodine nutritional status may become more significant. This also helps to explain the observed negative correlation between TSH and iodine nutritional status. Since our study is limited to cross-sectional data, further longitudinal studies are required to validate these findings.

In our analysis, we found that patients with relatively higher levels of Scr were more likely to benefit from RAIT. Published studies have also highlighted the importance of renal function in the metabolism of RAI and its retention time in the body (28, 43). Our analysis provides some evidence for a potential association between renal function and RAIT efficacy. Impaired renal function can slow the excretion of RAI, thereby prolonging its retention time in the body, which may enhance the localized cytotoxic effects of *β* radiation in thyroid tissue (43). However, it is important to note that patients with higher Scr levels in this cohort may also be influenced by other metabolic factors, and the additional risks associated with radiation exposure should not be overlooked. Therefore, further prospective clinical trials are necessary to better define the impact of Scr on RAIT efficacy and to explore the potential underlying mechanisms. Interestingly, we observed that a relatively higher Scr level within the normal physiological range may be associated with more favorable recovery of thyroid hormone levels posttreatment. This raised a hypothesis that dietary interventions, such as a moderate increase in protein intake, might influence treatment outcomes by modulating Scr levels. However, it remains essential to ensure that Scr levels are maintained within the normal range to avoid renal burden or other adverse effects. This potential mechanism is still speculative and warrants clarification through further clinical studies.

In this analysis, 25(OH)D was found to have no impact on the efficacy of RAIT or thyroid function. This finding contrasts with several studies that propose a potential role for vit D in modulating thyroid function following RAIT. Płazińska et al. found that vit D deficiency, particularly in smokers, affects the recovery of thyroid function in GH patients undergoing RAIT, especially leading to an increase in TRAb (24). In CD4^+^ T-cell responses, vit D directly inhibits the production of Th1 cytokines (IL-2 and IFN-c) and increases the production of Th2 cytokines (IL-4). The increase in Th2 cytokines helps reduce TRAb and alleviates the autoimmune response in patients with GH (24, 44, 45). In contrast, our study did not differentiate between smokers and nonsmokers, which may partly explain the lack of such an association. Furthermore, our study had a relatively short follow-up period after RAIT, and the potential immunomodulatory effects of vit D during long-term follow-up were not fully observed. The effects of 25(OH)D in RAIT still require further investigation.

Although the study found a partially significant correlation between liver function indicators (ALT and AST) and changes in thyroid function after RAIT, the changes in ALT and AST before and after RAIT were not significant. Therefore, even though liver function may play a role in other metabolic pathways, its specific impact on RAIT outcomes was not supported by the findings of this study (27).

In this study, we found no significant associations between thyroid weight, RAIU, smoking status, or iodine-rich food consumption and the efficacy of RAIT (**Supplementary Table S5**). These results are consistent with the study of Nishio et al., further underscoring the complexity and potential limitations of these variables in predicting RAIT efficacy (20).

Smoking has been repeatedly linked to higher relapse rates in GH, an increased risk of Graves ophthalmopathy (GO) following RAIT, and poorer GO treatment responses (46). Płazińska et al. also demonstrated that smokers had higher titers of thyroid-stimulating hormone receptor antibodies with or without concomitant vit D supplementation, suggesting that smoking may exacerbate the immunopathology and clinical course of GH (24). Nevertheless, we observed no significant relationship between smoking and RAIT efficacy in our cohort. This discrepancy may be attributed to immune-mediated pathways or clinical characteristics through which smoking influences treatment response, but which were not fully measured in our study. In addition, heterogeneity in treatment regimens, patient demographics, or other unaccounted confounding factors may also have contributed to the lack of observed association.

Although dietary iodine intake is recognized as a modulator of thyroid function, we did not detect a significant impact of an iodine‐rich diet on RAIT outcomes, in agreement with the findings of Nishio et al. (20). Interestingly, however, our data revealed that iodine nutritional status correlated differently with treatment efficacy at early versus late stages after RAIT. Whereas previous studies have treated iodine intake as a static variable that is considered to have a minimal impact on RAIT success, our observations suggest that the timing and dynamic adjustment of iodine consumption throughout the treatment course may be critical. We therefore hypothesize that modulating iodine intake in accordance with the RAIT phase could optimize therapeutic response. It should be noted that our analyses were confined to biomarker‐level data. The utility of dynamically tailored dietary iodine interventions to enhance RAIT efficacy remains to be validated in prospective clinical trials.

This study has several inherent limitations. First, the follow-up period was confined to 6 months after the initial RAIT. As a subset of patients who remained unresponsive after 6 months may have undergone a second RAIT, a 6-month observation period may be insufficient to fully capture rates of relapse or the long-term risk of hypothyroidism. Therefore, a prospective study with extended follow-up (e.g., ≥ 12 months) is warranted in the future to confirm our findings and to more fully assess the efficacy and safety of RAIT. Second, owing to the limitations of our clinical follow-up protocol, thyroid function assessment was confined to four biomarkers (TRAb, FT3, FT4, and TSH), therefore unable to fully characterize thyroid autoimmunity after RAIT. Future work should incorporate a broader range of immune markers to clarify how nutritional status and organ functional status affect immune dynamics during RAIT. Third, the cohort included in this study is from Northeast Asia, a region with high iodine levels, where dietary habits are characterized by high salt and seasoning intake. This may somewhat limit the generalizability of the findings to other populations. Moreover, factors such as sunlight exposure and lifestyle may affect 25(OH)D, which could contribute to discrepancies when comparing with results from previous studies.

## Conclusion

To the best of our knowledge, this is the first systematic analysis to investigate the potential impacts of iodine nutritional status, 25(OH)D, and liver and kidney function on therapeutic efficacy and thyroid function during RAIT in patients with hyperthyroid. The results indicated that age was a relatively influential factor affecting RAIT efficacy. Additionally, iodine nutritional status exhibited dynamic changes in its effect on TRAb and TSH at different time points, with SIC found to be more advantageous than UIC for monitoring iodine nutritional status. Scr was significantly associated with changes in thyroid function following RAIT. Within the normal range, higher Scr levels were potentially associated to decreases in FT3 and FT4, as well as increases in TSH. In contrast, ALT and AST did not show significant changes before and after RAIT and exhibited a weak correlation with thyroid function.

## Data Availability

The original contributions presented in the study are included in the article/**Supplementary Material**. Further inquiries can be directed to the corresponding author.
